# Knowledge, attitudes, and practices of esophageal cancer patients regarding pulmonary rehabilitation training

**DOI:** 10.3389/fpubh.2025.1647650

**Published:** 2025-11-03

**Authors:** Wei Wang, Wenqun Xing, Peinan Chen, Haibo Sun, Zongfei Wang, Yan Zheng, Yongkui Yu, Deming Zeng

**Affiliations:** ^1^Department of Thoracic Surgery, The Affiliated Cancer Hospital of Zhengzhou University & Henan Cancer Hospital, Zhengzhou, Henan, China; ^2^Department of Physical Medicine and Rehabilitation, Xiangya Hospital Central South University, Changsha, Hunan, China

**Keywords:** knowledge, attitude, practice, esophageal cancer, pulmonary rehabilitation, cross-sectional study

## Abstract

**Introduction:**

This study aimed to assess the knowledge, attitudes, and practices (KAP) of esophageal cancer patients concerning pulmonary rehabilitation training.

**Methods:**

A cross-sectional study was conducted at the Henan Cancer Hospital from July 1, 2024, to August 31, 2024. Data were collected through questionnaires that gathered demographic information and KAP scores.

**Results:**

A total of 530 esophageal cancer patients participated, including 197 (37.17%) regular smokers and 145 (27.36%) regular alcohol consumers. The mean ± SD scores were 7.78 ± 4.56 for knowledge (range: 0–20), 40.42 ± 4.66 for attitudes (range: 10–50), and 21.13 ± 3.08 for practices (range: 5–25). Correlation analyses showed positive relationships between knowledge and attitude scores (*r* = 0.335, *p* < 0.001), knowledge and practice scores (*r* = 0.323, *p* < 0.001), and attitude and practice scores (*r* = 0.567, *p* < 0.001). Structural Equation Modeling (SEM) indicated significant effects of knowledge on attitude (*β* = 0.420, *p* < 0.001) and attitude on practice (*β* = 0.711, *p* < 0.001).

**Conclusion:**

Esophageal cancer patients showed inadequate knowledge but positive attitudes and proactive practices regarding pulmonary rehabilitation. These findings highlight the need for targeted educational interventions to improve patient knowledge, enhancing overall engagement in rehabilitation practices.

## Introduction

Esophageal cancer ranks seventh in incidence and sixth in mortality among all malignancies globally, with a higher prevalence in men, particularly those aged 60 to 70 years (1). In 2020, there were approximately 604,000 new cases and 544,000 deaths worldwide, with significant regional variations in incidence and mortality. The highest incidence rates were observed in Eastern Asia and Southern and Eastern Africa, driven by specific risk factors such as tobacco use, alcohol consumption, hot beverage intake, and indoor air pollution. Projections indicate that the global burden of esophageal cancer will increase by over 50% from 2020 to 2040, reaching nearly 1 million new cases annually (2).

Esophagectomy is the cornerstone of treatment for resectable esophageal cancer; however, it carries higher morbidity and mortality rates compared to other gastrointestinal surgeries (3, 4). Although esophagectomy is crucial for treating esophageal cancer, the surgical procedure may compress the lungs and damage thoracic muscles, leading to postoperative pulmonary complications such as respiratory dysfunction, atelectasis, and even respiratory failure. These complications can severely impair patients’ quality of life and hinder recovery, further complicating postoperative treatments (5, 6). Pulmonary morbidity is a common complication following esophagectomy, with recent studies reporting that it still occurs in 16 to 23% of cases despite improvements (4, 7–9). Pulmonary complications are also a major cause of hospital mortality and may independently predict poorer long-term survival (10, 11). Pulmonary rehabilitation plays a vital role in mitigating these postoperative complications. Through interventions such as respiratory function training and guided physical exercises, pulmonary rehabilitation can effectively improve lung function, alleviate breathing difficulties, and enhance exercise tolerance after surgery. Long-term, systematic respiratory training has been widely recognized for its ability to improve lung function and reduce the incidence of complications following thoracic surgeries, including esophagectomy, ultimately improving patients’ overall survival and quality of life. From this perspective, incorporating pulmonary rehabilitation into postoperative care is increasingly being emphasized in clinical practice (5, 12).

KAP (Knowledge, Attitude, and Practice) theory emphasizes that knowledge is the foundation for behavior change, while attitudes and beliefs serve as the driving force behind such changes (13). According to KAP theory, behavior change progresses through three stages: acquiring knowledge, forming attitudes and beliefs, and finally, adopting practices and behaviors (14). However, knowledge alone does not automatically lead to behavior change; it must first alter perceptions, which in turn drive behavioral adjustments (15).

China is among the top five countries with the highest incidence of esophageal cancer, with Linxian in Henan Province and Cixian and Shexian in Hebei Province reporting the highest incidence rates globally (1, 16). While esophagectomy remains a critical treatment for resectable esophageal cancer, the high rates of postoperative pulmonary complications greatly affect patient recovery and long-term survival (17, 18). Therefore, understanding the KAP of esophageal cancer patients regarding pulmonary rehabilitation training is essential for developing targeted educational and intervention strategies. Effective strategies could improve rehabilitation outcomes by enhancing lung function, reducing pulmonary complications, and improving the patients’ quality of life following surgery. Given the importance of this issue, research focusing on this population is crucial for informing clinical practice and optimizing rehabilitation approaches.

Currently, there is a lack of KAP studies specifically addressing pulmonary rehabilitation in this patient population. Unlike previous KAP studies in oncology, this research specifically targets pulmonary rehabilitation among esophageal cancer patients and employs both conventional statistical analyses and structural equation modeling (SEM) to elucidate the interrelationships among knowledge, attitudes, and practices. This design provides new insights into behavioral factors influencing rehabilitation engagement and offers an evidence base for developing targeted educational strategies in this high-risk surgical population. This study aimed to assess the KAP of esophageal cancer patients concerning pulmonary rehabilitation training.

## Materials and methods

### Study design and participants

This cross-sectional study was conducted at the Henan Cancer Hospital from July 1, 2024, to August 31, 2024, involving esophageal cancer patients. This study was approved by the Ethic Committee of Henan Cancer Hospital (2024–282), and all participants provided written informed consent.

Inclusion Criteria: Patients diagnosed with esophageal cancer through clinical and auxiliary examinations, those eligible for radical esophageal cancer surgery, and those without a history of mental illness or communication barriers.

Exclusion Criteria: Patients who were uncooperative or demonstrated poor treatment adherence, pregnant or breastfeeding patients, those with congenital deformities or congenital diseases, patients with severe diseases of major organs or acute/chronic infections, and those with severe liver or kidney dysfunction or acute/chronic diseases.

Questionnaires were distributed to participants in both electronic and paper formats.

### Questionnaire introduction

The questionnaire was developed based on relevant guidelines and literature (19–21). Following its initial design, the questionnaire was revised according to feedback from three experts, including two rehabilitation specialists, and a pilot test was conducted with 30 participants. The questionnaire demonstrated a reliability coefficient of 0.911.

The final questionnaire, written in Chinese, comprised four dimensions with a total of 37 items: Basic Information (11 items), Knowledge Dimension (11 items, including a trap question as the 8th item to identify invalid responses), Attitude Dimension (10 items), and Practice Dimension (5 items). For statistical analysis, scores were assigned according to the number of response options. In the Knowledge Dimension, responses of “very familiar” were scored 2 points, “heard of” 1 point, and “not clear” 0 points, with a total possible score ranging from 0 to 20 points. In the Attitude Dimension, items A1-A5 (positive attitude) were scored from 5 points (“strongly agree”) to 1 point (“strongly disagree”), while items A6-A10 (negative attitude) were reverse-scored from 1 point (“strongly agree”) to 5 points (“strongly disagree”), resulting in a total possible score ranging from 10 to 50 points. In the Practice Dimension, responses ranged from 5 points (“always”) to 1 point (“never”), with a total possible score ranging from 5 to 25 points (Supplementary Questionnaire). A scoring threshold of greater than 70% was established for each dimension to define adequate knowledge, positive attitudes, and proactive practices (22).

This study was conducted at a single center. The questionnaires were distributed in both electronic format via the Wenjuanxing platform and paper format with QR codes in outpatient and inpatient settings. A total of 578 questionnaires were collected from willing participants. Of these, 35 were incomplete or involved mid-study withdrawals, and 10 were discarded due to careless or random responses. After excluding 1 questionnaire from a participant under 18 years old and 2 questionnaires with logical errors in the trap question, 530 valid questionnaires were included in the final analysis.

### Sample size calculation

The sample size was calculated using the formula for determining the minimum sample size in cross-sectional studies:


$$n={\left(\frac{Z_{1-\alpha /2}}{\delta }\right)}^{2}\times p\times (1-p)$$

Where *α* = 0.05.

$Z_{1-\alpha /2}=1.96$ .

*δ* = 0.05.

*p* = 0.5.

$n={\left(\frac{1.96}{0.05}\right)}^{2}\times 0.5\times (1-0.5)\approx 384$ .

This calculation resulted in a minimum required sample size of 384. Considering an anticipated effective questionnaire return rate of 80%, a minimum of 480 questionnaires were planned to be collected.

### Statistical methods

Descriptive statistics will be utilized for demographic data and KAP scores, with continuous data presented as means and standard deviations (SD), and categorical responses reported as *n* (%). Differences in knowledge (K), attitudes (A), and practices (P) scores across different demographic groups will be compared using t-tests for two-group comparisons and ANOVA for comparisons among three or more groups. Multivariate regression analysis will be performed with practice scores as the dependent variable to examine the relationships among demographic characteristics, knowledge, attitudes, and practices. Practice scores will be dichotomized based on 70% of the maximum score, and all relevant variables will be included in the regression model. *p*-values will be reported to three decimal places, with *p* < 0.05 considered statistically significant. Statistical analyses will be conducted using SPSS 26.0 and AMOS 26.0 (IBM, Armonk, NY, United States).

## Results

### Basic information on the population

Among the 530 esophageal cancer patients included in this study, 391 (73.77%) were male, with a mean age of 65.60 ± 7.82 years. A total of 389 participants (73.40%) had an educational level of middle school or below, 316 (59.62%) had a monthly per capita income of less than 2,000 yuan, 197 (37.17%) were regular smokers, 145 (27.36%) regularly consumed alcohol, and 397 (74.91%) currently had eating disorders. The mean ± SD scores for knowledge, attitude, and practice were 7.78 ± 4.56, 40.42 ± 4.66, and 21.13 ± 3.08, respectively. Analysis of demographic characteristics revealed that knowledge, attitude, and practice scores varied significantly by residence (*p* < 0.001, *p* = 0.009, *p* < 0.001), education level (*p* < 0.001, *p* < 0.001, *p* < 0.001), employment status (*p* = 0.001, *p* < 0.001, *p* = 0.004), and monthly per capita income (*p* < 0.001, *p* < 0.001, *p* < 0.001). Additionally, knowledge and attitude scores differed significantly by gender (*p* = 0.036, *p* = 0.002) and smoking status (*p* = 0.023, *p* = 0.005) (Table 1).

**Table 1:** Baseline characteristics and knowledge, attitude, practice (KAP) scores.

Variables	*N* (%)	Knowledge		Attitude		Practice	
		Mean ± SD	*P*	Mean ± SD	*P*	Mean ± SD	*P*
Total	530	7.78 ± 4.56		40.42 ± 4.66		21.13 ± 3.08	
Gender			0.036		0.002		0.135
Male	391 (73.77)	8.02 ± 4.76		40.79 ± 4.58		21.25 ± 3.09	
Female	139 (26.23)	7.08 ± 3.87		39.37 ± 4.74		20.80 ± 3.03	
Age (years old)	65.60 ± 7.82						
Residence			<0.001		0.009		<0.001
Rural	368 (69.43)	7.21 ± 4.50		40.02 ± 4.72		20.82 ± 3.15	
Urban	75 (14.15)	9.71 ± 4.89		41.67 ± 4.48		22.44 ± 2.37	
Suburban	87 (16.42)	8.48 ± 3.96		41.01 ± 4.36		21.34 ± 3.02	
Education			<0.001		<0.001		<0.001
Middle school and below	389 (73.40)	7.20 ± 4.34		39.86 ± 4.55		20.81 ± 3.01	
High school/technical school	95 (17.92)	8.54 ± 4.69		41.52 ± 4.66		21.72 ± 3.17	
Associate degree/bachelor’s degree and above	46 (8.68)	11.07 ± 4.52		42.87 ± 4.49		22.70 ± 2.85	
Employment status			0.001		<0.001		0.004
Employed	48 (9.06)	9.42 ± 4.99		42.35 ± 4.30		22.27 ± 2.66	
Retired	87 (16.42)	8.76 ± 4.88		41.49 ± 5.12		21.62 ± 3.52	
Other	395 (74.53)	7.36 ± 4.36		39.94 ± 4.51		20.89 ± 2.99	
Monthly per capita income (yuan)			<0.001		<0.001		<0.001
<2000	316 (59.62)	6.92 ± 4.51		39.28 ± 4.66		20.43 ± 3.16	
2000–5,000	168 (31.70)	8.52 ± 3.95		41.93 ± 3.79		21.98 ± 2.64	
<5,000	46 (8.68)	10.91 ± 5.12		42.67 ± 5.20		22.87 ± 2.55	
Marital status			0.241		0.720		0.532
Married	506 (95.47)	7.83 ± 4.57		40.43 ± 4.65		21.15 ± 3.11	
Other	24 (4.53)	6.71 ± 4.13		40.08 ± 4.92		20.75 ± 2.45	
Do you smoke regularly?			0.023		0.005		0.105
Yes	197 (37.17)	8.36 ± 4.71		41.16 ± 4.07		21.42 ± 3.15	
No	333 (62.83)	7.43 ± 4.43		39.98 ± 4.93		20.97 ± 3.03	
Do you drink alcohol regularly?			0.161		0.130		0.691
Yes	145 (27.36)	8.23 ± 4.78		40.92 ± 4.48		21.22 ± 3.23	
No	385 (72.64)	7.61 ± 4.46		40.23 ± 4.72		21.10 ± 3.03	
How long have you been diagnosed with esophageal cancer?			0.499		0.780		0.911
Less than 1 year	527 (99.43)	7.79 ± 4.56		40.42 ± 4.66		21.13 ± 3.08	
1 year or more	3 (0.57)	6.00 ± 2.65		39.67 ± 4.93		21.33 ± 3.21	
Do you currently have any eating disorders?			0.690		0.486		0.927
Yes	397 (74.91)	7.82 ± 4.64		40.50 ± 4.68		21.14 ± 3.09	
No	133 (25.09)	7.64 ± 4.30		40.17 ± 4.61		21.11 ± 3.07	

### Knowledge attitude practice

In the knowledge dimension, the three questions with the highest proportion of participants selecting “Not clear” were: “Do you know that during pulmonary rehabilitation training, the intensity should be moderate to high (where the patient feels slightly breathless and fatigued but can continue) to achieve optimal benefits?” (K6) with 43.77%, “Do you know that pulmonary rehabilitation training is a personalized comprehensive intervention conducted after a thorough assessment of the patient’s condition?” (K3) with 43.40%, and “Do you know that if symptoms such as cough, sputum production, or worsening of breathing difficulties occur due to a cold or other reasons, pulmonary rehabilitation training should only be resumed after at least 2 weeks of symptom relief?” (K7) with 42.26% (Supplementary Table S1).

Regarding attitude, 11.13% agreed that “pulmonary rehabilitation training is less important than other preoperative preparations because it takes too long to show effects” (A6), 7.36% agreed that “even if the pulmonary rehabilitation program is strictly followed, it might not be effective, so there is no need for strict adherence” (A7), and 6.04% agreed that “their family might not cooperate with or support them in carrying out pulmonary rehabilitation training” (A9) (Supplementary Table S2).

For the practice dimension, 16.79% sometimes and 3.40% seldom shared knowledge about esophageal cancer and the preoperative pulmonary rehabilitation program with friends and relatives to gain their support (P4), 13.40% sometimes and 5.09% seldom sought to learn relevant knowledge (P1), and 11.70% sometimes and 1.32% seldom maintained a positive attitude toward esophageal cancer treatment and pulmonary rehabilitation training, believing that this attitude would ultimately benefit them (P5) (Supplementary Table S3).

### Correlations between KAP

Further correlation analysis revealed positive correlations between knowledge scores and attitude scores (*r* = 0.335, *p* < 0.001), between knowledge scores and practice scores (*r* = 0.323, *p* < 0.001), and between attitude scores and practice scores (*r* = 0.567, *p* < 0.001) (Table 2).

**Table 2:** Correlation analysis.

Correlation	Knowledge	Attitude	Practice
Knowledge	1		
Attitude	0.335 (*P* < 0.001)	1	
Practice	0.323 (*P* < 0.001)	0.567 (*P* < 0.001)	1

### Factors associated with KAP

Multivariate logistic regression analysis showed that age (OR = 0.945, 95% CI: [0.904, 0.988], *p* = 0.012) and a monthly per capita income of 2,000–5,000 yuan (OR = 0.353, 95% CI: [0.143, 0.870], *p* = 0.024) were independently associated with knowledge (Table 3). Additionally, a monthly per capita income of 2,000–5,000 yuan (OR = 2.848, 95% CI: [1.189, 6.823], *p* = 0.019) was independently associated with a positive attitude (Table 4). Moreover, the attitude score (OR = 1.298, 95% CI: [1.202, 1.401], *p* < 0.001) was independently associated with proactive practice (Table 5).

**Table 3:** Multivariate logistic regression analysis-knowledge dimension.

Variables	Univariate analysis		Multivariate analysis	
	OR (95%CI)	*P*	OR (95%CI)	*P*
Gender				
Male	Ref.		Ref.	
Female	0.407 (0.188, 0.881)	0.023	0.567 (0.235, 1.373)	0.209
Age (years old)	0.941 (0.911, 0.973)	<0.001	0.945 (0.904, 0.988)	0.012
Residence				
Rural	Ref.		Ref.	
Urban	4.416 (2.327, 8.379)	<0.001	1.921 (0.712, 5.182)	0.197
Suburban	1.758 (0.838, 3.685)	0.136	1.420 (0.615, 3.278)	0.412
Education				
Middle school and below	Ref.		Ref.	
High school/technical school	1.968 (0.980, 3.950)	0.057	1.689 (0.664, 4.297)	0.271
Associate degree/bachelor’s degree and above	7.277 (3.584, 14.775)	<0.001	3.154 (0.791, 12.574)	0.104
Employment status				
Employed	Ref.		Ref.	
Retired	0.516 (0.219, 1.215)	0.130	1.800 (0.572, 5.664)	0.315
Other	0.237 (0.114, 0.494)	<0.001	1.200 (0.350, 4.110)	0.772
Monthly per capita income (yuan)				
<2000	Ref.		Ref.	
2000–5,000	1.049 (0.542, 2.032)	0.886	0.353 (0.143, 0.870)	0.024
<5,000	6.275 (3.064, 12.851)	<0.001	0.980 (0.292, 3.291)	0.973
Marital status				
Married	Ref.			
Other	0.336 (0.045, 2.533)	0.290		
Do you smoke regularly?				
Yes	1.743 (1.012, 3.004)	0.045	1.173 (0.618, 2.226)	0.625
No	Ref.		Ref.	
Do you drink alcohol regularly?				
Yes	1.546 (0.874, 2.737)	0.135		
No	Ref.			
How long have you been diagnosed with esophageal cancer?				
Less than 1 year	Ref.			
1 year or more	–	0.999		
Do you currently have any eating disorders?				
Yes	0.981 (0.526, 1.826)	0.951		
No	Ref.			

**Table 4:** Multivariate logistic regression analysis-attitude dimension.

Variables	Univariate analysis		Multivariate analysis	
	OR (95%CI)	*P*	OR (95%CI)	*P*
Knowledge score	1.084 (1.012, 1.160)	0.021	1.061 (0.988, 1.140)	0.106
Gender				
Male	Ref.			
Female	0.640 (0.349, 1.175)	0.150		
Age (years old)	0.960 (0.923, 1.000)	0.049	0.996 (0.952, 1.042)	0.850
Residence				
Rural	Ref.			
Urban	2.287 (0.794, 6.582)	0.125		
Suburban	1.739 (0.715, 4.233)	0.223		
Education				
Middle school and below	Ref.			
High school/technical school	1.095 (0.512, 2.343)	0.815		
Associate degree/bachelor’s degree and above	1.643 (0.487, 5.538)	0.423		
Employment status				
Employed	Ref.			
Retired	0.377 (0.078, 1.820)	0.225		
Other	0.375 (0.088, 1.604)	0.186		
Monthly per capita income (yuan)				
<2000	Ref.		Ref.	
2000–5,000	3.429 (1.503, 7.823)	0.003	2.848 (1.189, 6.823)	0.019
<5,000	1.565 (0.533, 4.595)	0.415	1.057 (0.330, 3.384)	0.925
Marital status				
Married	Ref.			
Other	0.751 (0.216, 2.607)	0.651		
Do you smoke regularly?				
Yes	2.105 (1.076, 4.116)	0.030	1.838 (0.919, 3.675)	0.085
No	Ref.		Ref.	
Do you drink alcohol regularly?				
Yes	1.145 (0.592, 2.212)	0.688		
No	Ref.			
How long have you been diagnosed with esophageal cancer?				
Less than 1 year	Ref.			
1 year or more	0.214 (0.019, 2.404)	0.212		
Do you currently have any eating disorders?				
Yes	1.111 (0.582, 2.122)	0.749		
No	Ref.			

**Table 5:** Multivariate logistic regression analysis-practice dimension.

Variables	Univariate analysis		Multivariate analysis	
	OR (95%CI)	*P*	OR (95%CI)	*P*
Knowledge score	1.166 (1.088, 1.249)	<0.001	1.065 (0.986, 1.151)	0.108
Attitude score	1.329 (1.239, 1.425)	<0.001	1.298 (1.202, 1.401)	<0.001
Gender				
Male	Ref.			
Female	0.957 (0.529, 1.732)	0.884	0.968 (0.922, 1.016)	0.189
Age (years old)	0.907 (0.869, 0.948)	<0.001		
Residence				
Rural			Ref.	
Urban	6.006 (1.430, 25.226)	0.014	2.573 (0.539, 12.271)	0.236
Suburban	1.426 (0.674, 3.018)	0.353	0.908 (0.388, 2.123)	0.824
Education				
Middle school and below	Ref.			
High school/technical school	2.026 (0.891, 4.608)	0.092		
Associate degree/bachelor’s degree and above	3.546 (0.835, 15.056)	0.086		
Employment status				
Employed	Ref.			
Retired	0.300 (0.064, 1.416)	0.128		
Other	0.300 (0.071, 1.274)	0.103		
Monthly per capita income (yuan)				
<2000	Ref.		Ref.	
2000–5,000	4.847 (2.155, 10.902)	<0.001	1.840 (0.722, 4.692)	0.202
<5,000	9.483 (1.280, 70.268)	0.028	3.526 (0.397, 31.339)	0.258
Marital status				
Married	Ref.			
Other	3.212 (0.426, 24.203)	0.258		
Do you smoke regularly?				
Yes	1.312 (0.748, 2.303)	0.344		
No	Ref.			
Do you drink alcohol regularly?				
Yes	0.934 (0.521, 1.673)	0.818		
No	Ref.			
How long have you been diagnosed with esophageal cancer?				
Less than 1 year	Ref.			
1 year or more	-	0.999		
Do you currently have any eating disorders?				
Yes	1.118 (0.617, 2.027)	0.713		
No	Ref.			

### Interactions between KAP

**Figure 1:**
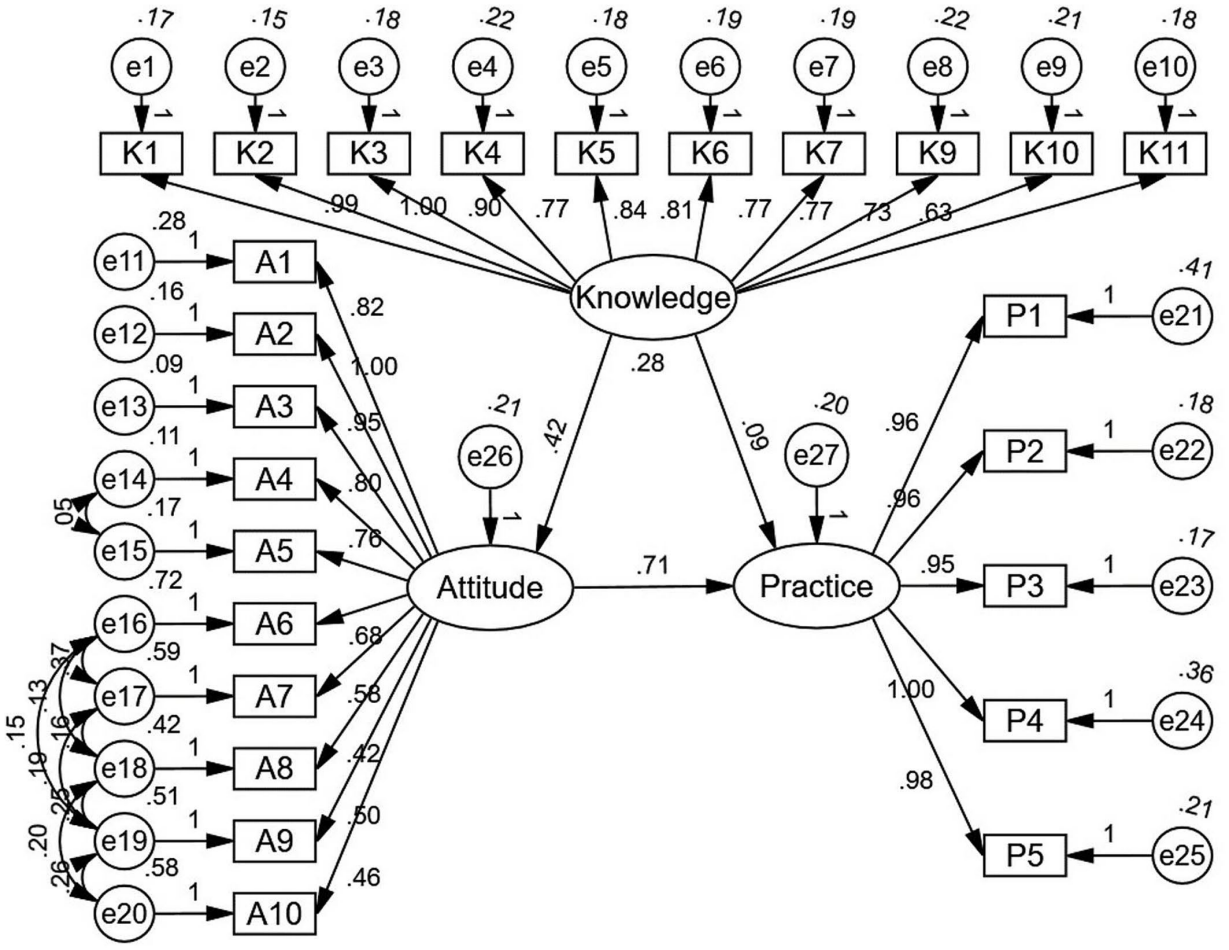
SEM analysis result. This figure presents the path model for Knowledge, Attitude, and Practice (KAP) variables, displaying the relationships among various indicators. The standardized path coefficients are shown along each arrow, indicating the strength of the relationship between constructs. Circles labeled e1–e27 represent error terms associated with each observed variable, while the elliptical shapes represent the latent variables of Knowledge, Attitude, and Practice. Direct effects are denoted by arrows, with significant pathways indicating strong relationships between Knowledge and Attitude (0.42), Attitude and Practice (0.71), and Knowledge and Practice (0.28). Indicator variables (K1-K11 for Knowledge, A1-A10 for Attitude, and P1-P5 for Practice) show their respective factor loadings, demonstrating their contribution to the latent constructs.

The Structural Equation Modeling (SEM) analysis demonstrated good model fit with the following indices: RMSEA = 0.060, IFI = 0.924, TLI = 0.913, and CFI = 0.924 (Supplementary Table S1). The results indicated a significant direct effect of knowledge on attitude (*β* = 0.420, *p* < 0.001) and of attitude on practice (*β* = 0.711, *p* < 0.001), while the direct effect of attitudes on practice was not significant (*β* = 0.092, *p* = 0.073) (Supplementary Table S2 and Figure 1).

## Discussion

Esophageal cancer patients demonstrated inadequate knowledge but maintained positive attitudes and proactive practices concerning pulmonary rehabilitation training. These findings suggest the need for targeted educational interventions to enhance patients’ knowledge, which may further strengthen their attitudes and practices in clinical settings.

The results of this study show that while esophageal cancer patients demonstrated positive attitudes and proactive practices toward pulmonary rehabilitation training, their knowledge was notably inadequate. This finding is consistent with other studies, which have similarly reported low awareness, skepticism about the necessity, and limited acceptance of pulmonary rehabilitation among COPD patients in China (23). Additionally, there remains a significant gap in understanding and referral practices for pulmonary rehabilitation among Chinese respiratory physicians, further limiting access for patients with chronic respiratory diseases (24). The positive attitudes and proactive behaviors observed in this study suggest that patients are generally willing to engage in rehabilitation. However, the lack of knowledge may hinder them from fully benefiting from these programs. This gap between attitude and knowledge highlights the critical need for targeted educational interventions to ensure that patients are adequately informed and able to effectively participate in pulmonary rehabilitation. In addition to individual knowledge and socioeconomic disparities, difficulties in follow-up and limited accessibility to medical personnel may also hinder effective pulmonary rehabilitation among cancer patients. In real-world clinical settings, many patients have limited opportunities to communicate with their physicians due to short consultation times and heavy workloads in oncology departments. Consequently, important issues such as medication side effects, symptom management, and the rationale behind rehabilitation training are often insufficiently discussed. Establishing regular follow-up systems and enhancing doctor–patient communication could therefore be crucial to improving patients’ understanding, adherence, and overall participation in pulmonary rehabilitation programs. Furthermore, the rise of artificial intelligence–based medical platforms and telemedicine systems has greatly improved patients’ ability to follow up and understand their treatment processes. These technologies facilitate continuous communication between patients and healthcare providers, enhance access to educational resources, and enable personalized rehabilitation guidance even outside hospital settings. Such innovations have shown promising applications in esophageal surgery and postoperative care, providing new opportunities for improving patient outcomes (25–27).

The findings from correlation analysis, multivariate logistic regression, and SEM all indicate significant interconnections. These results were supported by the SEM analysis, which demonstrated that knowledge had a significant direct effect on attitude (*β* = 0.420, *p* < 0.001) and attitude on practice (*β* = 0.711, *p* < 0.001). However, the direct effect of knowledge on practice was not significant (*β* = 0.092, *p* = 0.073), suggesting that attitudes may act as a mediator between knowledge and practice. This highlights the importance of not only improving patients’ knowledge but also ensuring that their attitudes are aligned with evidence-based practices to encourage positive behavioral outcomes (28, 29). In this model, knowledge appears to influence practice primarily through its effect on attitudes rather than through a direct pathway. This finding suggests that simply increasing patients’ knowledge about pulmonary rehabilitation may not be sufficient unless it is accompanied by changes in their beliefs and motivation toward participation. Educational efforts that connect factual understanding with perceived personal benefit may therefore play a key role in strengthening this indirect pathway.

When examining the individual variables, several significant associations were identified. For gender, male patients exhibited significantly higher knowledge (*p* = 0.036) and attitude scores (*p* = 0.002) compared to female patients. However, no significant difference was found in practice scores (*p* = 0.135). This could indicate that while men may be more informed and maintain a positive outlook toward pulmonary rehabilitation, both genders face similar barriers when it comes to implementing these practices. Residence was another important factor, with urban and suburban patients scoring higher in knowledge (*p* < 0.001), attitude (*p* = 0.009), and practice (*p* < 0.001) compared to rural patients. This may be attributed to better access to healthcare resources and educational materials in urban and suburban areas. Rural patients often face challenges such as limited access to healthcare services and lower health literacy, which could explain their lower scores across all three dimensions (30, 31).

Educational level was strongly associated with KAP scores, where patients with higher education levels demonstrated significantly better knowledge (*p* < 0.001), attitudes (*p* < 0.001), and practices (*p* < 0.001). This is consistent with existing literature indicating that education is a key determinant of health literacy and health-related behaviors (32, 33). The multivariate logistic regression analysis supported this, showing that higher education was independently associated with better knowledge and attitudes, which subsequently influenced practice scores. Employment status also showed significant differences in all KAP dimensions. Employed and retired patients scored higher in knowledge (*p* = 0.001), attitude (*p* < 0.001), and practice (*p* = 0.004) compared to those in other employment categories. This could be due to greater social support and access to information among employed and retired individuals. Interestingly, multivariate analysis indicated that employment status was not an independent predictor of KAP outcomes, suggesting that other factors, such as income and education, might mediate these relationships (34, 35).

Monthly per capita income was another significant variable, with higher income being associated with better knowledge (*p* < 0.001), attitudes (*p* < 0.001), and practices (*p* < 0.001). This association was further confirmed by multivariate logistic regression, which showed that a monthly income of 2,000–5,000 yuan was independently associated with better knowledge (OR = 0.353, 95% CI: [0.143, 0.870], *p* = 0.024) and attitudes (OR = 2.848, 95% CI: [1.189, 6.823], *p* = 0.019). Higher income likely provides better access to healthcare resources, educational materials, and a supportive environment, facilitating better health outcomes (36).

Interestingly, significant differences in KAP were not observed across marital status, smoking, or alcohol consumption for practice scores, despite differences in knowledge and attitudes. For example, regular smokers had higher knowledge (*p* = 0.023) and attitude (*p* = 0.005) scores compared to non-smokers, yet no significant difference was observed in practice scores (*p* = 0.105). This lack of difference in practice might be attributed to external factors such as limited access to resources or support for behavioral changes, suggesting that improving knowledge alone may not be sufficient to translate into better practices without addressing these underlying barriers (37).

The distribution of responses across the knowledge, attitude, and practice dimensions indicates several areas where esophageal cancer patients exhibit inadequate understanding, particularly regarding the principles and importance of pulmonary rehabilitation. A significant portion of the patients were unclear about key aspects of pulmonary rehabilitation, such as its components, intensity, and the necessity for personalization based on specific health conditions. For instance, nearly half of the patients were unfamiliar with the importance of moderate to high-intensity training during pulmonary rehabilitation, and many did not recognize that rehabilitation could continue effectively outside of a hospital setting. These findings are consistent with similar studies in which patients often show limited understanding of complex medical regimens, leading to suboptimal engagement and outcomes (38). On the other hand, the attitude and practice dimensions revealed generally positive responses, with the majority of patients expressing interest and willingness to participate in rehabilitation, although a small subset harbored misconceptions or doubts about its effectiveness.

Given these knowledge gaps, targeted educational interventions are essential. First, tailored educational materials should be developed to clearly explain the specific benefits of pulmonary rehabilitation, emphasizing the role of high-intensity training and the flexibility of performing these exercises outside the hospital. These materials could include visual aids, simplified language, and practical examples that resonate with patients’ everyday experiences. In addition, hands-on workshops or interactive sessions led by healthcare professionals could enhance patient understanding and retention of this information (39–41).

Considering the significant disparities observed in knowledge across different demographic groups, specific strategies should be implemented to address these gaps. For example, rural patients and those with lower educational levels demonstrated notably poorer knowledge scores. For these groups, community-based programs that leverage local healthcare workers could be particularly effective. Such programs might include home visits or small group sessions that offer personalized education and support, thereby overcoming barriers related to health literacy and access. Moreover, employing digital platforms like mobile apps tailored to deliver bite-sized, easy-to-understand information on pulmonary rehabilitation could reach a broader audience, particularly among younger or tech-savvy patients (42, 43).

To improve the more challenging aspects, such as the misunderstanding about the necessity of strict adherence to rehabilitation plans, motivational interviewing techniques could be integrated into routine care. This approach has been shown to effectively change health behaviors by aligning the intervention with patients’ personal values and goals (44, 45). Additionally, addressing concerns related to the potential for harm or the perceived burden of rehabilitation on family dynamics should be a priority. Structured family counseling sessions, where both patients and their families receive clear guidance on the benefits and practical aspects of rehabilitation, could alleviate these concerns and foster a supportive environment.

This study has several limitations. First, as a cross-sectional study, it captures only a snapshot of the participants’ KAP at a single point in time, limiting the ability to assess changes over time or causality. Future research may include longitudinal or interventional designs to explore how knowledge, attitudes, and practices evolve over time and to examine whether targeted educational measures can lead to measurable behavioral improvements. Second, the data were collected using self-reported questionnaires, which may introduce response bias, particularly in sensitive areas such as smoking and alcohol consumption. Because the responses were self-reported, some participants might have provided socially desirable answers or understated certain behaviors. Although the questionnaire included a control item to identify inconsistent responses, self-reporting bias cannot be completely ruled out. Third, the study was conducted at a single cancer hospital, which may limit the generalizability of the findings to other populations or settings. Because the participants were all recruited from one institution, the findings should be interpreted with some caution. Differences in healthcare resources, patient education, and rehabilitation practices in other regions may lead to somewhat different outcomes. Future studies involving several centers could help verify whether these patterns hold in broader contexts.

In conclusion, esophageal cancer patients demonstrated inadequate knowledge, positive attitudes, and proactive practices concerning pulmonary rehabilitation training. These findings highlight the need for targeted educational interventions to enhance patients’ knowledge, which may further strengthen their attitudes and practices toward pulmonary rehabilitation, ultimately improving their overall treatment outcomes.

## Data Availability

The original contributions presented in the study are included in the article/Supplementary material, further inquiries can be directed to the corresponding author.
