# Supplementary material for: Knowledge, attitudes, and practices of esophageal cancer patients regarding pulmonary rehabilitation training
Source: Front Public Health. 2025 Nov 3;13:1647650. doi: 10.3389/fpubh.2025.1647650 (PMC12620307; doi:10.3389/fpubh.2025.1647650)
Supplement: Supplementary file 1 [file Table_1.docx]

**Table S1 Knowledge dimension**

| **For the following statements, please indicate your level of understanding:** | **Very familiar**  **N (%)** | **Heard of it**  **N (%)** | **Not clear**  **N (%)** |
| --- | --- | --- | --- |
| 1. You know that esophageal cancer patients often require thoracotomy during surgery, which may lead to a reduction in restrictive ventilation function preoperatively, so preoperative pulmonary rehabilitation training is necessary. | 106 (20.00) | 296 (55.85) | 128 (24.15) |
| 1. You know that preoperative pulmonary rehabilitation training for esophageal cancer patients can restore normal cyclic deep breathing, improve respiratory function, and is an effective method to prevent preoperative pulmonary complications. | 71 (13.40) | 278 (52.45) | 181 (34.15) |
| 1. You know that pulmonary rehabilitation training is a personalized comprehensive intervention conducted after a thorough assessment of the patient’s condition. | 49 (9.25) | 251 (47.36) | 230 (43.40) |
| 1. You know that the main components of pulmonary rehabilitation training include patient assessment, exercise training, health education, nutritional intervention, and psychosocial support. | 58 (10.94) | 305 (57.55) | 167 (31.51) |
| 1. You know that the principle of pulmonary rehabilitation training is similar to normal physical exercise, aiming to exceed daily life loads without causing discomfort. | 44 (8.30) | 283 (53.40) | 203 (38.30) |
| 1. You know that during pulmonary rehabilitation training, the training intensity should be moderate to high (where the patient feels slightly breathless and fatigued but can continue) to gain optimal benefits. | 36 (6.79) | 262 (49.43) | 232 (43.77) |
| 1. You know that if symptoms such as cough, sputum production, or worsening of breathing difficulties occur due to a cold or other reasons, pulmonary rehabilitation training should be resumed only after at least two weeks of symptom relief. | 32 (6.04) | 274 (51.70) | 224 (42.26) |
| 1. You know that if you have concurrent cardiovascular or cerebrovascular diseases, pulmonary rehabilitation training should be tailored to your specific condition. | 47 (8.87) | 282 (53.21) | 201 (37.92) |
| 1. You know that the training plan for pulmonary rehabilitation needs to be formulated by professional medical staff. | 69 (13.02) | 336 (63.40) | 125 (23.58) |
| 1. You know that pulmonary rehabilitation training does not necessarily have to be conducted in a hospital; training at home or in the community can be equally effective. | 78 (14.72) | 374 (70.57) | 78 (14.72) |

**Table S2 Attitude dimension**

|  | **Strongly agree N (%)** | **Agree N(%)** | **Neutral N (%)** | **Disagree N (%)** | **Strongly disagree N (%)** |
| --- | --- | --- | --- | --- | --- |
| 1. You are very interested in learning about the causes, treatment methods, and intervention plans related to esophageal cancer, including pulmonary rehabilitation training. (P) | 200 (37.74) | 269 (50.75) | 58 (10.94) | 2 (0.38) | 1 (0.19) |
| 1. You believe that esophageal cancer is a very serious illness that requires personal attention and a serious approach to the preoperative pulmonary rehabilitation plan provided by medical staff. (P) | 193 (36.42) | 280 (52.83) | 55 (10.38) | 2 (0.38) | 0 |
| 1. You trust the preoperative pulmonary rehabilitation plan provided by medical staff. (P) | 215 (40.57) | 290 (54.72) | 25 (4.72) | 0 | 0 |
| 1. You believe that any discomfort experienced during pulmonary rehabilitation training should be addressed promptly by seeking medical attention. (P) | 203 (38.30) | 314 (59.25) | 13 (2.45) | 0 | 0 |
| 1. You believe that monitoring details such as heart rate, breathing, blood oxygen saturation, and blood pressure is important during pulmonary rehabilitation training. (P) | 203 (38.30) | 312 (58.87) | 12 (2.26) | 2 (0.38) | 1 (0.19) |
| 1. You think that pulmonary rehabilitation training is less important than other preoperative preparations because it takes too long to show effects. (N) | 10 (1.89) | 59 (11.13) | 132 (24.91) | 259 (48.87) | 70 (13.21) |
| 1. You believe that even if you strictly follow the pulmonary rehabilitation plan provided by medical staff, it might still be ineffective, so strict adherence is not necessary. (N) | 9 (1.70) | 39 (7.36) | 115 (21.70) | 310 (58.49) | 57 (10.75) |
| 1. You think that pulmonary rehabilitation exercises might cause other harm to the body or even worsen the symptoms of esophageal cancer. (N) | 8 (1.51) | 16 (3.02) | 66 (12.45) | 360 (67.92) | 80 (15.09) |
| 1. You believe that your family might not cooperate with or support you in carrying out pulmonary rehabilitation training. (N) | 9 (1.70) | 32 (6.04) | 81 (15.28) | 332 (62.64) | 76 (14.34) |
| 1. You believe that undergoing pulmonary rehabilitation training before surgery might make it apparent to friends and family that you are ill, and you would prefer not to be subject to discrimination or be a burden to your family, so you do not want to participate. (N) | 9 (1.70) | 29 (5.47) | 86 (16.23) | 336 (63.40) | 70 (13.21) |

**Table S3 Practice dimension**

|  | **Always N (%)** | **Often N (%)** | **Sometimes N (%)** | **Rarely N (%)** | **Never N (%)** |
| --- | --- | --- | --- | --- | --- |
| 1. You are willing to learn about the causes, treatment methods, and reasons for preoperative pulmonary rehabilitation training for esophageal cancer, including specific plans and related knowledge. (P) | 201 (37.92) | 229 (43.21) | 71 (13.40) | 27 (5.09) | 2 (0.38) |
| 1. You are willing to strictly follow the preoperative pulmonary rehabilitation plan recommended by medical staff. (P) | 226 (42.64) | 245 (46.23) | 50 (9.43) | 9 (1.70) | 0 |
| 1. You are willing to strictly follow any other pulmonary rehabilitation plans provided by medical staff after surgery. (P) | 225 (42.45) | 244 (46.04) | 55 (10.38) | 6 (1.13) | 0 |
| 1. You are willing to share knowledge about esophageal cancer and the preoperative pulmonary rehabilitation plan with family and friends to gain their support. (P) | 191 (36.04) | 229 (43.21) | 89 (16.79) | 18 (3.40) | 3 (0.57) |
| 1. You are willing to approach esophageal cancer treatment and pulmonary rehabilitation training with a positive attitude and believe that this attitude will ultimately benefit you. (P) | 237 (44.72) | 223 (42.08) | 62 (11.70) | 7 (1.32) | 1 (0.19) |

**Table S4 Model Fit**

| **Indicators** | **Reference** | **Actual** |
| --- | --- | --- |
| CMIN/DF | 1-3: Excellent, 3-5: Good | 2.927 |
| RMSEA | <0.08: Good | 0.060 |
| IFI | >0.8: Good | 0.924 |
| TLI | >0.8: Good | 0.913 |
| CFI | >0.8: Good | 0.924 |

**Table S5 Structural Equation Modeling (SEM) Path Coefficients and Significance Levels**

|  |  |  | **Estimate** | **P** |
| --- | --- | --- | --- | --- |
| Attitude | <--- | Knowledge | 0.420 | <0.001 |
| Practice | <--- | Attitude | 0.711 | <0.001 |
| Practice | <--- | Knowledge | 0.092 | 0.073 |
| K1 | <--- | Knowledge | 0.987 | <0.001 |
| K2 | <--- | Knowledge | 1.000 |  |
| K3 | <--- | Knowledge | 0.903 | <0.001 |
| K4 | <--- | Knowledge | 0.766 | <0.001 |
| K5 | <--- | Knowledge | 0.844 | <0.001 |
| K6 | <--- | Knowledge | 0.811 | <0.001 |
| K7 | <--- | Knowledge | 0.766 | <0.001 |
| K9 | <--- | Knowledge | 0.773 | <0.001 |
| K10 | <--- | Knowledge | 0.727 | <0.001 |
| K11 | <--- | Knowledge | 0.630 | <0.001 |
| A10 | <--- | Attitude | 0.458 | <0.001 |
| A9 | <--- | Attitude | 0.498 | <0.001 |
| A8 | <--- | Attitude | 0.423 | <0.001 |
| A7 | <--- | Attitude | 0.577 | <0.001 |
| A6 | <--- | Attitude | 0.675 | <0.001 |
| A5 | <--- | Attitude | 0.765 | <0.001 |
| A4 | <--- | Attitude | 0.804 | <0.001 |
| A3 | <--- | Attitude | 0.952 | <0.001 |
| A2 | <--- | Attitude | 1.000 |  |
| A1 | <--- | Attitude | 0.825 | <0.001 |
| P1 | <--- | Practice | 0.961 | <0.001 |
| P2 | <--- | Practice | 0.960 | <0.001 |
| P3 | <--- | Practice | 0.949 | <0.001 |
| P4 | <--- | Practice | 1.000 |  |
| P5 | <--- | Practice | 0.984 | <0.001 |
